# Gastric schwannoma: a case report

**DOI:** 10.1093/jscr/rjae181

**Published:** 2024-03-27

**Authors:** Ognen Kostovski, Gjorgji Trajkovski, Gligor Ristovski, Slavica Kostadinova Kunovska, Irena Kostovska

**Affiliations:** University Clinic of Digestive Surgery, Clinical Center “Mother Theresa”, 1000 Skopje, Republic of North Macedonia; University Clinic of Digestive Surgery, Clinical Center “Mother Theresa”, 1000 Skopje, Republic of North Macedonia; Institute of Pathology, Faculty of Medicine, Ss. Cyril and Methodius University in Skopje, 50 Divizija 6,1000 Skopje, Republic of North Macedonia; Institute of Pathology, Faculty of Medicine, Ss. Cyril and Methodius University in Skopje, 50 Divizija 6,1000 Skopje, Republic of North Macedonia; Department of Medical and Experimental Biochemistry, Faculty of Medicine, Ss. Cyril and Methodius University in Skopje, 50 Divizija 6, 1000 Skopje, Republic of North Macedonia

**Keywords:** gastric schwannoma, immunohistochemistry, rare gastric tumors

## Abstract

Gastric schwannomas are rare mesenchymal tumors that arise from the intestinal nerve plexuses. They present with nonspecific symptoms and are often discovered incidentally. We present the case of a 68-year-old patient who had been suffering from abdominal discomfort for 6 months. After a complete examination, including abdominal computed tomography and upper gastrointestinal endoscopy, we discovered a submucosal gastric lesion with benign gross features without evidence of lymph node or metastatic involvement. He underwent an open laparotomy. Final pathohistological and immunohistochemically identification of the surgical specimen established the diagnosis of benign schwannoma. Considering the excellent prognosis of the tumor, no adjuvant treatment was suggested other than simple clinical monitoring every 6 months. Despite the accessibility of advanced endoscopy and imaging techniques, the diagnosis of gastric schwannoma is rarely made preoperatively. In the latter case, the best treatment is still complete excision with wide margins.

## Introduction

Schwannoma (or neurilema) is a nerve tumor originating from Schwann cells. Schwannomas can form in any nerve, often in the head or neck and much less often in the gastrointestinal tract. The most common site of gastrointestinal schwannomas is the stomach, which accounts for only 0.2% of gastric neoplasms. Gastrointestinal schwannomas are a rare entity of gastrointestinal mesenchymal tumors, and only a few clinical cases have been reported [1]. However, physicians do not widely recognize gastrointestinal schwannomas, hence, gastrointestinal schwannomas remain difficult to distinguish accurately from other gastric submucosal tumors preoperatively. It is generally a benign tumor with an excellent prognosis after complete resection [2]. We report a case of a 68-year-old male patient with gastric schwannoma who was admitted to a university. The patient provided written informed consent for this record to be published. Ethics approval was waived for this study because it was a retrospective case report without impacting patient care.

### Case report

**Figure 1:**
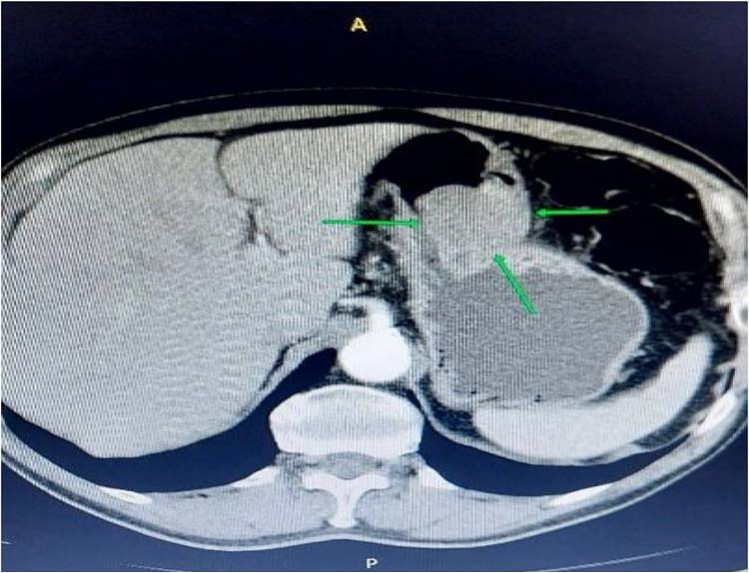
Contrast-enhanced abdominal computerized tomography. The axial section presents a tumor mass measuring 55*85 mm in diameter.

A 68-year-old man was referred to the gastroenterology department because of lower abdominal discomfort for the past 6 weeks. He was asymptomatic for gastric lesions, and a physical examination of the abdomen showed no evidence of tenderness or a palpable mass. The medical history was unremarkable. He had no previous surgical treatments or other illnesses. Routine blood tests were unremarkable. The patient underwent a contrast-enhanced computed tomography (CT) scan of the abdomen and was found to have a significant submucosal mass, ~5–7 cm, toward the antral region and along the greater curvature of the gastric body (Fig. 1).

**Figure 2:**
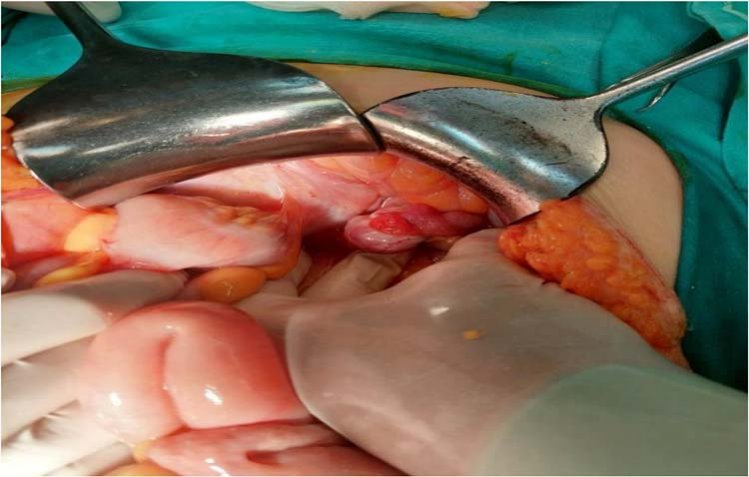
Supraumbilical median laparotomy.

The patient was counseled on surgical intervention with a provisional diagnosis of gastrointestinal stromal tumor (GST). Supraumbilical median laparotomy was performed, with excision parietis ventriculi tangentialis cum excision tumoris in toto (Fig. 2). Macroscopic examination of the surgical specimen revealed the formation of well-defined nodules between the mucosa and gastric serosa, ~6 cm in diameter.

**Figure 3:**
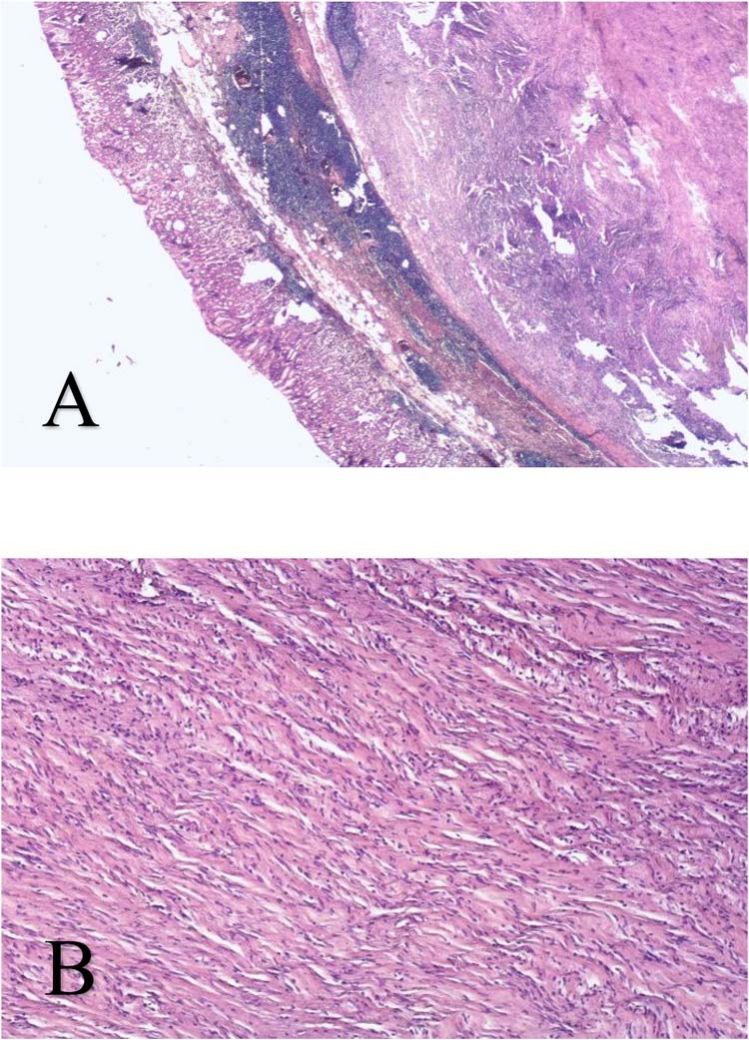
Spindle cell neoplasm arranged in interwoven connective tissue separated by a myxoid or hyalinized stroma - HeEo × 5 (A), HeEo × 40 (B).

**Figure 4:**
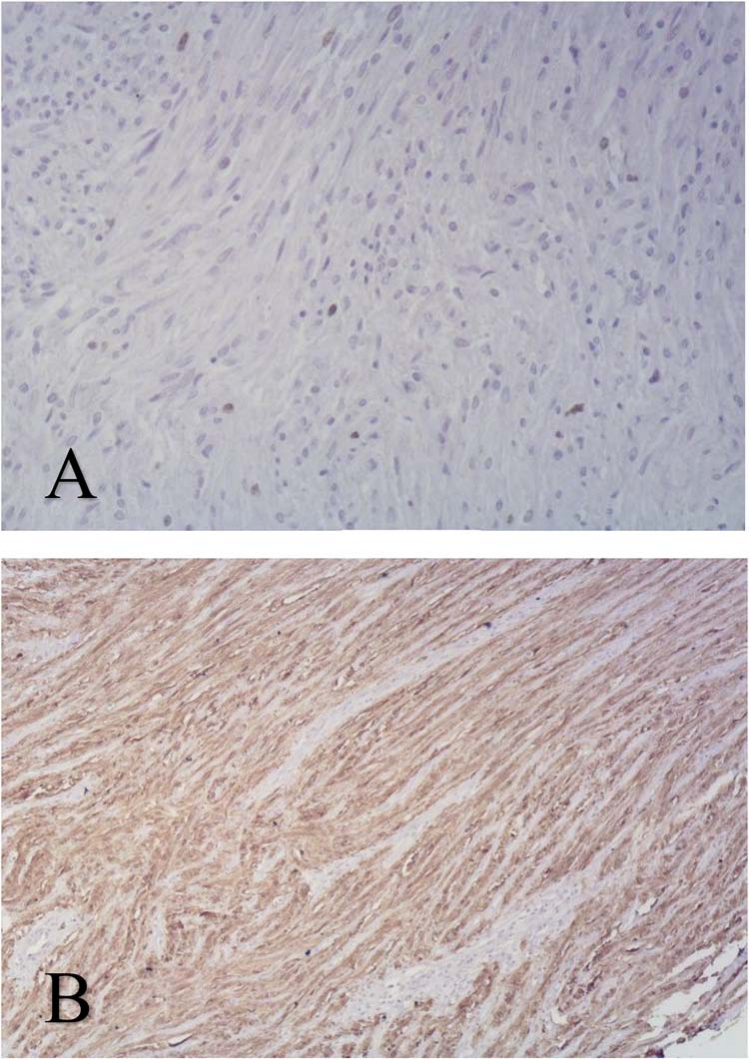
Immunohistochemical staining of biopsy specimen Ki67 × 100 (A), S100 protein×100 (B).

Microscopic analysis of the resected and H&E-stained specimens showed a spindle cell tumor in interwoven connective tissue separated by a myxoid or hyalinized stroma consistent with schwannoma (Fig. 3). Neoplastic cells were immunoreactive for S-100 protein. Ki-67 proliferation index is < 1% (Fig. 4). Histopathological features and immunohistochemical staining pattern proved the diagnosis of gastric schwannoma.

The post-operative period was uneventful, and he was discharged from our hospital on day eight after surgery, without complaints at a follow-up after 1 month.

## Discussion

Gastrointestinal schwannoma is a rare gastrointestinal mesenchymal tumor first described by Daimaru et al. in 1988 [3]. They are thought to arise from the plexus of the intestinal wall, unlike common schwannomas, which arise from peripheral nerves in the skin, connective tissue, and internal organs. Gastric schwannoma can occur at any age but is most often seen in the fifth and sixth decades, with a predominance in women. Although many schwannomas are discovered incidentally, they cause nonspecific symptoms such as pain, epigastric mass, and bleeding. Malignant transformation of gastric schwannoma is extremely rare. Only a few cases have been described in the literature [4]. Endoscopy and imaging techniques cannot distinguish gastric mesenchymal tumors because they all present as isolated submucosal masses. Most are of submucosal origin and are located in the body of the stomach, ranging in size from 0.5 to 11 cm in previously reported cases. In this case, the tumor was located in the region anti-ad curvature majority of the stomach. It measured 8.5 × 5.5 × 5 cm. Since the definitive preoperative diagnosis is challenging to establish, as well as possible complications such as bleeding or stenosis are difficult to prevent, surgical resection needs to be considered the primary strategy of treatment for patients with gastric schwannoma [5]. Schwannoma diagnosis is based on tissue histology, further confirmed by immunohistochemical markers. Immunohistochemistry shows strong nuclear and cytoplasmic staining patterns for S100 protein and vimentin and consistent negativity for CD-117, CD-34, and SMA [6, 7]. Differentiating gastric schwannomas from GST can be difficult preoperatively, as imaging studies such as sonography, endoscopy, and CT scans have not revealed specific features unique to these tumors. Gastric schwannomas have been reported to be uniquely different from other schwannomas in that they exhibit homogeneous attenuation on CT and that degenerative changes such as cystic changes are rare. A homogeneous healing pattern can help distinguish gastric schwannomas from GSTs, which often show heterogeneous healing due to degenerative changes [7]. Before the identification of the S-100 antigen in gastric schwannoma and GST, these tumors were often classified as leiomyoma, leiomyosarcoma, or gastrointestinal autonomic nervous system tumors. Currently, complete surgical resection of the tumor is the only effective treatment method, and the prognosis after tumor resection is excellent.
